# Temporal metabolic response to mRNA COVID-19 vaccinations in oncology patients

**DOI:** 10.1007/s12149-021-01675-8

**Published:** 2021-08-31

**Authors:** Pooja Advani, Saranya Chumsri, Tanmayi Pai, Zhuo Li, Akash Sharma, Ephraim Parent

**Affiliations:** 1 Department of Oncology, Mayo Clinic Florida, Jacksonville, USA; 2 Department of Internal Medicine, Mayo Clinic Florida, Jacksonville, USA; 3 Department of Biostatistics, Mayo Clinic Florida, Jacksonville, USA; 4 Department of Radiology, Mayo Clinic Florida, 4500 San Pablo Rd S., Jacksonville, FL 32224 USA

**Keywords:** COVID-19, Vaccine, FDG

## Abstract

**Background:**

mRNA COVID-19 vaccines are known to provide an immune response seen on FDG PET studies. However, the time course of this metabolic response is unknown. We here present a temporal metabolic response to mRNA COVID-19 vaccination in oncology patients undergoing standard of care FDG PET.

**Methods:**

262 oncology patients undergoing standard of care FDG PET were included in the analysis. 231 patients had at least one dose of mRNA COVID-19 vaccine while 31 patients had not been vaccinated. The SUVmax of the lymph nodes ipsilateral to the vaccination was compared to the contralateral to obtain an absolute change in SUVmax (ΔSUVmax).

**Results:**

ΔSUVmax was more significant at shorter times between FDG PET imaging and COVID-19 mRNA vaccination, with a median ΔSUVmax of 2.6 (0–7 days), 0.8 (8–14 days), and 0.3 (> 14 days), respectively.

**Conclusion:**

Consideration should be given to performing FDG PET at least 2 weeks after the COVID-19 vaccine.

**Supplementary Information:**

The online version contains supplementary material available at 10.1007/s12149-021-01675-8.

## Introduction

With rapid and widespread rollout of the mRNA COVID-19 vaccinations [BNT162b2 (Pfizer-BioNTech vaccine) and mRNA-1273 (Moderna vaccine)], transient increased 2-deoxy-2[^18^F]fluoro-D-glucose (FDG) uptake in normal or enlarged ipsilateral axillary, supraclavicular, and cervical lymph nodes after vaccination has been reported in patients undergoing positron emission tomography (PET) examinations [1, 2]. This vaccine-induced lymphadenopathy may create FDG PET/CT interpretation challenges, particularly in cancer patients, affecting assessment of cancer staging and treatment response. While vaccine-induced lymphadenopathy has been described with other vaccines such as influenza [3, 4], vaccine-induced lymphadenopathy appears to be more prominent with mRNA COVID-19 vaccines. The mRNA COVID-19 vaccines are reported to be more immunogenic than traditional vaccines [5], which may influence the extent and duration of vaccine-induced lymphadenopathy on imaging. Recent literature has shed light on the confounding effects of vaccine-induced lymphadenopathy and proposed timelines for consideration of performing PET/CT examinations in relation to COVID-19 vaccine administration. However, the timeline of response and the associated differences in maximum standardized uptake value (ΔSUVmax) between ipsilateral vaccinated axillary/subpectoral lymph nodes and contralateral lymph node chains is relatively unknown. The goal of this study is to examine the temporal impact of COVID-19 vaccine on the ΔSUVmax results obtained from FDG PET/CT and FDG PET/MRI scan in patients who received at least 1 dose of an mRNA COVID-19 vaccine compare compared to those that did not receive an mRNA COVID-19 vaccine.

## Materials and methods

A total of 262 patients, who underwent FDG PET/CT scan for work-up of cancer, cancer staging, or monitoring of therapy response at Mayo Clinic between 02/23/2021 and 04/16/2021 were included in this prospective analysis. Information regarding COVID-19 vaccine manufacturer, date of vaccination, site of administration, and patients symptoms (current and at time of vaccination) was collected via intake questionnaires given to patients by technologists performing the scan. Patient age, sex, race, and blood sugar at time of FDG PET were obtained from the medical record as well as the type of cancer and treatments. The study has been approved by the institutional review board, (IRB # 21-001818) and all subjects provided verbal consent and the need for written informed consent was waived. Visual and semi-quantitative analysis of metabolic response to the mRNA COVID-19 vaccine was determined by two board-certified nuclear radiologists. Patients with PET or CT evidence of lymph node metastatic involvement in the ipsilateral or contralateral axillary/sub-pectoral lymph node chains on prior FDG PET/CT were excluded from the analysis.

Categorical variables were summarized as frequency (percentage) and continuous variables were reported as median (range). Kruskal–Walis test was used to compare continuous variables among patients with different time intervals between vaccine and PET scan while Wilcoxon rank-sum test was used to compare continuous variables between groups with and without vaccine, as well as the pairwise comparison between patients in two different time intervals between vaccine and PET scan. Chi-squared test was used to compare categorical variables between groups. Ipsilateral lymph node size and SUVmax were compared to the contralateral axillary/sub-pectoral lymph node chains to obtain an absolute difference in SUVmax (ΔSUVmax). ΔSUVmax was calculated as the SUVmax in the vaccinated arm for vaccinated patients (or either arm for the not vaccinated patients)—SUVmax in the contralateral arm. All tests were two-sided with *p* value < 0.05 considered statistically significant. To account for multiple comparison, Bonferroni correction was used for the pairwise comparison between different time intervals. Linear regression models were used to identify univariable and multivariable predictors for the between-arms difference in SUV max difference. The analysis was done using R3.6.2 [6].

## Results

285 oncology patients undergoing standard of care FDG PET/CT or FDG PET/MRI, and who had prior FDG PET studies for comparison, were initially included in this study. 23 of these patients were excluded due to PET or CT evidence of known metastatic disease in the axillary/sub-pectoral lymph node chains on the prior FDG PET/CT examination. Median age of the remaining 262 patients was 70 (range 20–94) years and 231 patients had at least one dose of COVID-19 vaccine before the PET scan (median age: 71 years) while 31 patients did not receive the vaccine (median age: 59 years). Median blood sugar of the 31 unvaccinated patients was 104 mg/dL (range 77–214 mg/dL) and the median blood sugar of the 231 patients that were vaccinated was 103 mg/dL (range 68–213 mg/dL). Of the vaccinated and non-vaccinated patients, 51% and 48% were male, respectively, and 76% of non-vaccinated and 91% of vaccinated patients were Caucasian.

Of the 231 vaccinated patients, 113 (47%) were noted to have visually increased FDG uptake in the ipsilateral axillary/sub-pectoral lymph nodes in the PET scan compared to the contralateral. When stratified by time since vaccination, 70% (23/33) of patients who received the vaccine between 0 and 7 days prior to PET had visually evident uptake, which dropped to 55% (21/38) at 8–14 days and 44% (71/160) when greater than 2 weeks since vaccination. There was no substantial difference between patients undergoing FDG PET/CT > 2 weeks after vaccination (> 14 days) compared to those that waited more than 4 weeks (> 28 days) with 40% (31/78) of patients in this last group demonstrating visually appreciated ipsilateral FDG uptake.

**Fig. 1:**
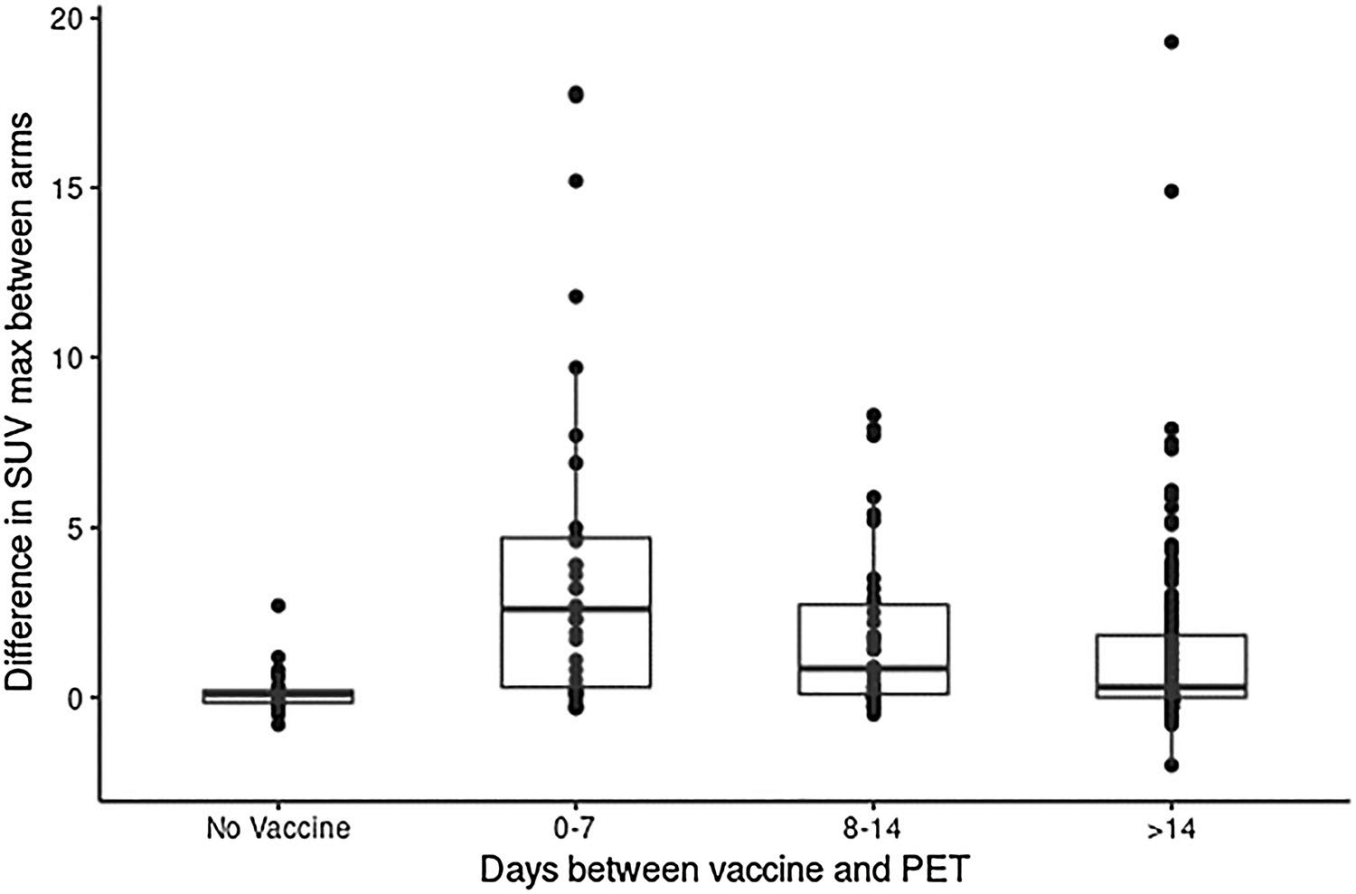
Box plot diagram of FDG PET ΔSUVmax values [vaccinated arm for vaccinated patients (or either arm for the not vaccinated patients)—SUVmax in the contralateral arm] in vaccinated and non-vaccinated patients] and days between vaccinations

**Fig. 2:**
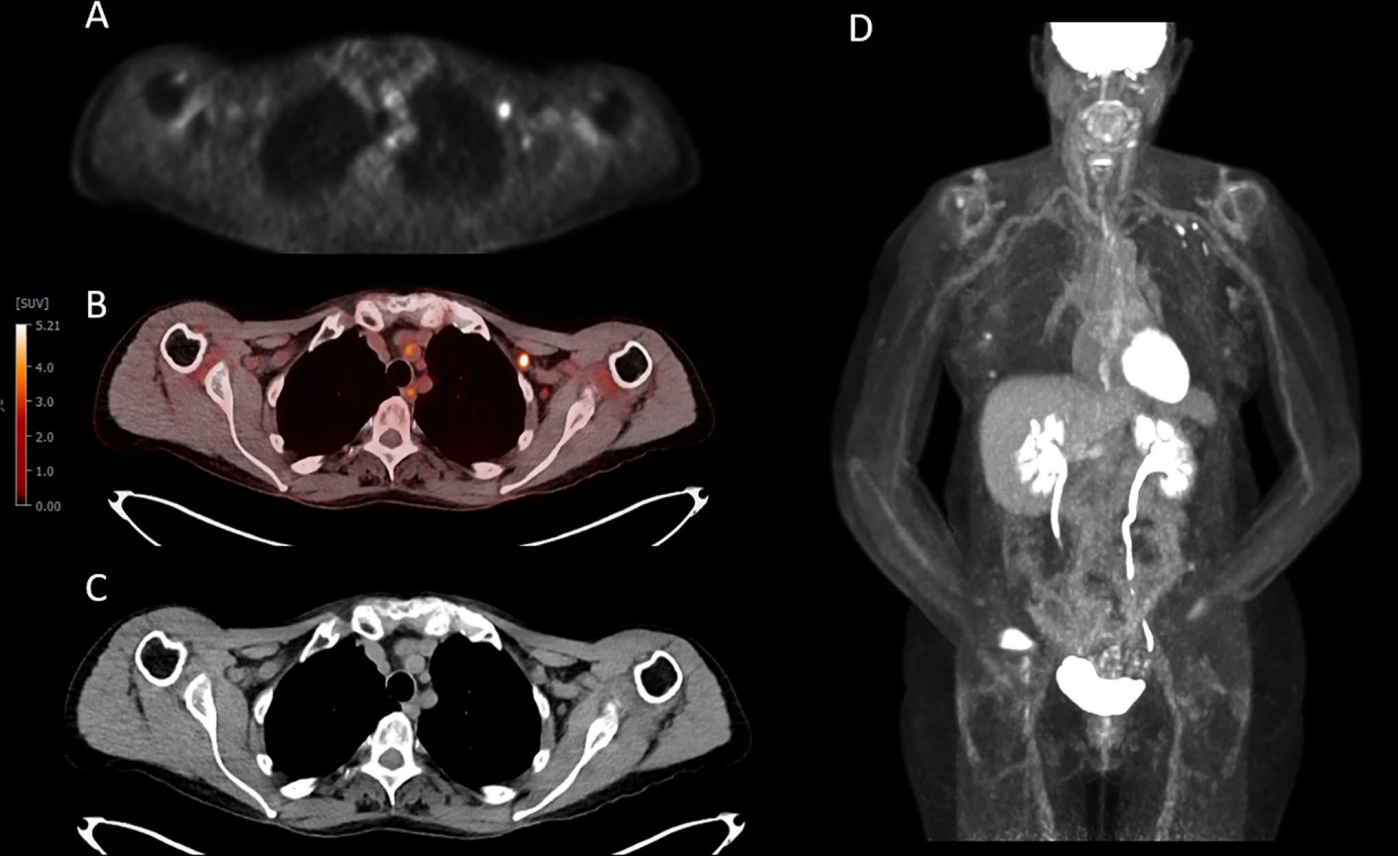
Selected images from serial FDG PET/CT examinations for a patient who received 2 doses of Moderna vaccination, with the second dose approximately 1 month before initial imaging. Trans-axial FDG PET (**A**) fused FDG PET/CT (**B**) transaxial non-contrast CT image (**C**) and FDG PET MIP (**D**) show two non-enlarged but hypermetabolic left axillary lymph nodes, ipsilateral to the COVID-19 vaccination sites in the left arm. ΔSUVmax was 6.7 and 6.8, in the anterior and posterior nodes, respectively

Semi-quantitative analysis confirmed visual findings with a median ΔSUVmax of 0.1 for patients without vaccine versus 0.4 for all patients with vaccine (*p* < 0.001, Table 1) regardless of time since vaccination. The greatest ΔSUVmax for an unvaccinated patient was 2.7. Time between COVID-19 vaccine and PET scan was associated with statistically significant changes in ΔSUVmax but remained greater than non-vaccinated patients at all time points. ΔSUVmax was greater at shorter time intervals between the dates of the COVID-19 vaccination with a median ΔSUVmax of 2.6 (0–7 days), 0.8 (8–14 days), and 0.3 (> 14 days), respectively (Fig. 1 and Table 2). Of the 160 patients that received a mRNA COVID-19 vaccination greater than 2 weeks prior to FDG PET, 12 patients demonstrated a ΔSUVmax > 5.0 (7.5%; Fig. 2). Additional quantitative analysis found non-significant differences in ΔSUVmax in vaccinated patients when comparing those patients that received vaccine > 14 days from FDG PET examination as compared to > 28 days (Supplemental). After adjusting for the presence of symptoms, ΔSUVmax retained greater significance at shorter intervals and the presence or absence symptoms were not found to be an independent variable for ΔSUVmax. Interestingly, of the 262 patients analyzed, only 5 were found to have enlarged axillary/sub-pectoral lymph nodes with a short axis > 1.0 cm and 4 of 5 reported symptoms including sore arm and flu-like symptoms. Also, 4 of the 5 received the mRNA vaccine within 7 days (range 3–7 days) of the FDG PET examination and had an average ΔSUVmax of 8.1 (range 3.2–17.7). The remaining patient with an enlarged axillary lymph node received the COVID-19 vaccination 30 days prior and had a ΔSUVmax of 1.7. Sub-analysis of blood sugar, age, race and gender found minor associations with ΔSUVmax; however, time from vaccine retained the most significant variable of FDG uptake (Supplemental).

**Table 1:** Between-arms difference in SUV max (ΔSUVmax) based on receipt of vaccine

	*N* (*N* = 31)	*Y* (*N* = 231)	Total (*N* = 262)	*p* value
Age				< 0.001
*N*	31	231	262	
Median (range)	59.00 (20.00, 92.00)	71.00 (20.00, 94.00)	70.00 (20.00, 94.00)	
Gender				0.78
F	16 (51.6%)	113 (48.9%)	129 (49.2%)	
M	15 (48.4%)	118 (51.1%)	133 (50.8%)	
Race				0.013
Non-Caucasian	7 (24.1%)	20 (8.9%)	27 (10.7%)	
Caucasian	22 (75.9%)	204 (91.1%)	226 (89.3%)	
Between-arms difference in SUV max				< 0.001
*N*	31	231	262	
Median (range)	0.10 (− 0.80, 2.70)	0.40 (− 2.00, 19.30)	0.30 (− 2.00, 19.30)	
Blood sugar				1.00
*N*	31	225	256	
Median (range)	104.00 (77.00, 14.00)	103.00 (68.00,213.00)	103.50 (68.00, 14.00)	
SUV/blood sugar				0.065
*N*	30	225	255	
Median (range)	0.01 (0.00, 0.05)	0.01 (0.00, 0.29)	0.01 (0.00, 0.29)

**Table 2:** Patient characteristics and ΔSUVmax according to days from vaccinations

	0–7 (*N* = 33)	8–14 (*N* = 38)	> 14 (*N* = 160)	*p* value
Days from vaccine				< 0.001
*N*	33	38	160	
Median (range)	5.0 (0.0, 7.0)	11.5 (8.0, 14.0)	28.0 (15.0, 93.0)	
ΔSUVmax				< 0.001
*N*	33	38	160	
Median (range)	2.6 (− 0.3, 17.8)	0.8 (− 0.5, 8.3)	0.3 (− 2.0, 19.3)	
Dose				< 0.001
1	14 (42.4%)	17 (44.7%)	26 (16.2%)	
2	19 (57.6%)	21 (55.3%)	134 (83.8%)	
Maker				0.023
MODERNA	20 (60.6%)	13 (35.1%)	94 (59.5%)	
PFIZER	13 (39.4%)	24 (64.9%)	64 (40.5%)	
Symptoms				0.068
No	28 (84.8%)	35 (92.1%)	153 (95.6%)	
Yes	5 (15.2%)	3 (7.9%)	7 (4.4%)

## Discussion

**Fig. 3:**
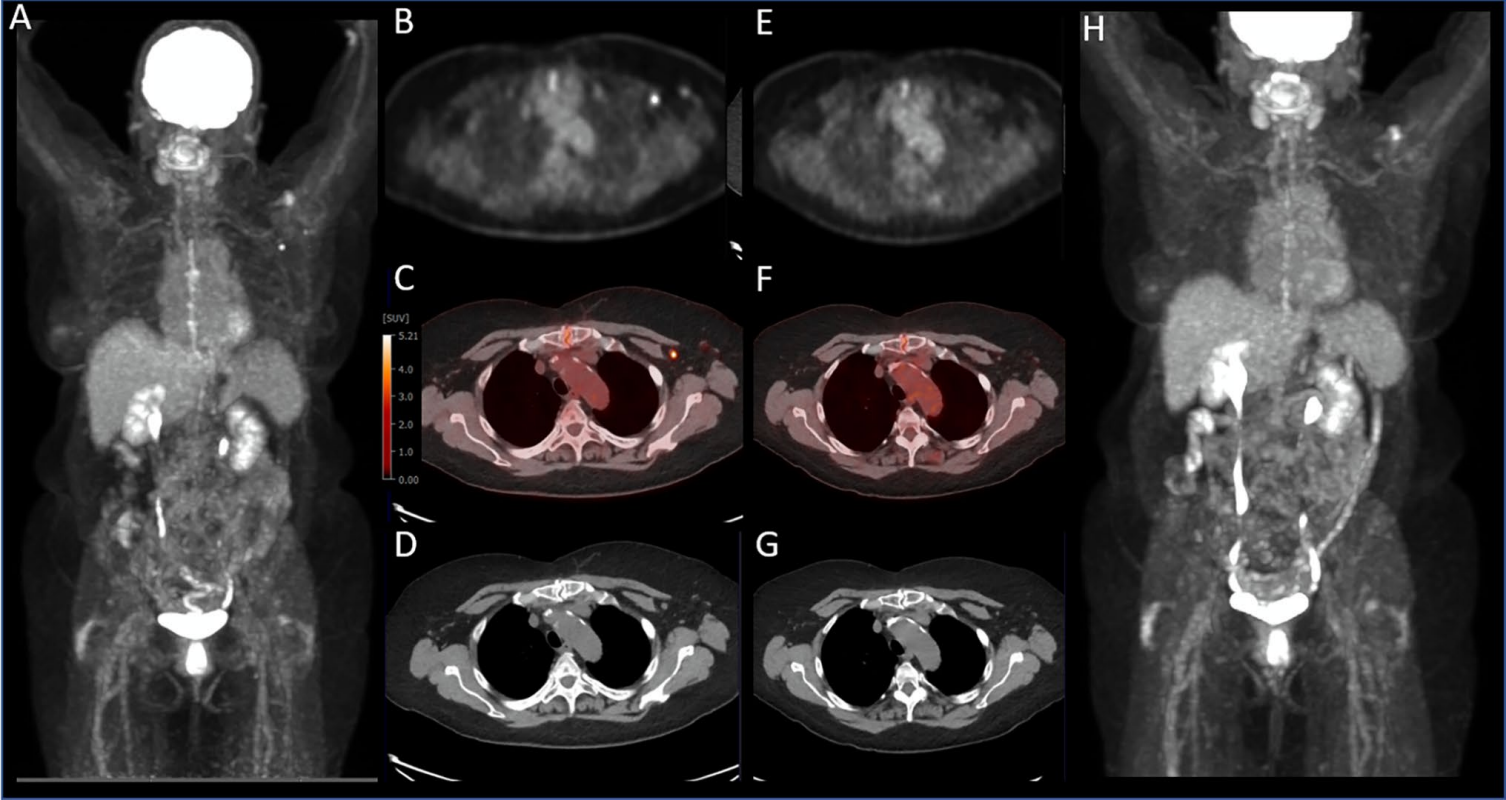
Selected images from serial FDG PET/CT examination for a patient who received 1 dose of Pfizer vaccination 11 days before initial imaging, as well as a second FDG PET/CT approximately 3 months later for clinical oncologic follow-up, with interval administration of the second dose of COVID-19 vaccination. FDG PET MIP (**A**) trans-axial FDG PET (**B**) fused FDG PET/CT (**C**) and trans-axial non-contrast CT images show a non-enlarged but hypermetabolic left axillary lymph node; ΔSUVmax = 5.9. On the subsequent evaluation, FDG PET MIP (**H**) trans-axial FDG PET (**E**) fused FDG PET/CT (**F**) and trans-axial non-contrast CT images FDG PET (**G**) images at matching levels show resolution of FDG uptake in the same-sized lymph node, now seen only on the CT image

Based on previous experience with routine vaccinations, vaccine-related nodal FDG uptake typically occurs within 7 days of vaccination and generally subsides by 12–14 days [7]. Studies have suggested performing PET/CT at least 2 weeks after vaccination in cancer patients for which interpretation is anticipated to be potentially impacted by the vaccination, though some have suggested to wait 4–6 weeks after vaccination [1]. Our data demonstrate a progressive decrease in SUV max after the first week from vaccine, and the lowest ΔSUVmax being seen after 2 weeks from vaccination, with non-substantial changes between patients waiting either > 2 weeks or > 4 weeks after vaccination. However, it should be noted that even after 2 weeks, there is a statistically significant ΔSUVmax for vaccinated patients compared to non-vaccinated patients, with a few outlying patients demonstrating markedly increased FDG uptake (Fig. 1). This is in line with the reported late-term metabolic response (7–10 weeks) after mRNA vaccination [8]. It has been postulated that symptomatic patients would have a greater radiographic response. However, our results demonstrate that self-reported symptoms do not affect the likelihood of having metabolically active nodal findings. Additionally, it should be noted that the vast majority of patients that received the vaccine did not have lymphadenopathy > 1.0 cm on the short axis despite many having increased FDG uptake (Fig. 3). This discrepancy between metabolic response (FDG PET) and lymphadenopathy has been previously reported and can be considered a hallmark of mRNA vaccination response and may help guide the interpreting physician to distinguish between a pronounced COVID-19 vaccination response and true nodal metastatic disease [9].

## Conclusion

Due to the relative recent and novel mRNA COVID-19 vaccinations, radiologists and oncologists are facing a diagnostic challenge in deciphering axillary, cervical and sub-pectoral lymphadenopathy in patients with active cancer or those that are undergoing work-up for cancer. Based on our study, consideration should be given to performing FDG PET/CT at least 2 weeks after the COVID-19 vaccine, if it does not interfere with the care of the patient. However, our findings also demonstrate that a small number of patients will continue to have a durable metabolic response which persists even after 2 weeks. Further studies regarding the optimal timing, and recommendations for follow-up of abnormal FDG PET/CT scan after COVID vaccine are needed.

## Supplementary Information

Below is the link to the electronic supplementary material.

Supplementary file1 (DOCX 59 KB)
