# Supplementary material for: Temporal metabolic response to mRNA COVID-19 vaccinations in oncology patients
Source: Ann Nucl Med. 2021 Aug 31;35(11):1264–9. doi: 10.1007/s12149-021-01675-8 (PMC8406005; doi:10.1007/s12149-021-01675-8)

# Statistical Analysis Method

## Categorical variables were summarized as frequency (percentage) and continuous variables were reported as median (range) . Kruskal-Walis test was used to compare continuous variables among patients with different time intervals between vaccine and PET scan while Wilcoxon rank sum test was used to compare continuous variables between groups with and without vaccine, as well as the pairwise comparison between patients in two different time intervals between vaccine and PET scan. Chi-squared test was used to compare categorical variables beetween groups. The between-arm difference in SUV max was calcualted as the SUV max in the vaccinated arm for vaccined aptients - SUV max in the contralateral arm. All tests were two-sided with p value <0.05 considered statistically significant. To account for multiple comparison, Bonferroni correction was used for the pairwise comparion between different time intervals. Linear regression models were used to identify univariable and multivariable predictors for the between-arm difference in SUV max differenc. The analysis was done using R3.6.2 (R Core Team (2019). R: A language and environment for statistical computing. R Foundation for Statistical Computing, Vienna, Austria).

# Brief description of results

## A total of 262 patients’ data were included in the analysis. The median between-arm difference in SUV max was 0.1 for patients without vaccine vs 0.4 for patients with vaccine (p<0.001, table 1). Further more, days between vaccine and PET scan was also assoicated with the between-arm difference in SUV max, the difference was bigger when the time interval between the two dates was smaller (table 2). After adjusting for age, gender, race, blood sugar and if symptoms were present, the impact of vaccine, and days from vaccine were still statistically significant. Compared to patients without vaccine, patient had vaccine >14 days, 8-14 days and 0-7 days ago were in average 1.8, 1.9 and 4.7 higher in between-arm difference in SUV max respectively (Table 4A; all p<0.05).

# Supplemental Table 1A. Vaccine data and between-arm difference in SUV max by days from vaccine

| Var.1 | 0-7 (N=33) | 8-14 (N=38) | >14 (N=160) | p value | p value 0-7 vs 8-14 days | p value 8-14 vs >14 days | p value 0-7 vs >14 days |
| --- | --- | --- | --- | --- | --- | --- | --- |
| Age |  |  |  | 0.275 | 0.576 | 0.113 | 0.532 |
| - N | 33 | 38 | 160 |  |  |  |  |
| - Median (Range) | 69.00 (27.00, 90.00) | 69.00 (32.00, 88.00) | 72.00 (20.00, 94.00) |  |  |  |  |
| Gender |  |  |  | 0.938 | 0.727 | 0.878 | 0.772 |
| - F | 17 (51.5%) | 18 (47.4%) | 78 (48.8%) |  |  |  |  |
| - M | 16 (48.5%) | 20 (52.6%) | 82 (51.2%) |  |  |  |  |
| Race |  |  |  | 0.270 | 0.684 | 0.350 | 0.126 |
| - Non-white | 5 (15.2%) | 4 (11.8%) | 11 (7.0%) |  |  |  |  |
| - White | 28 (84.8%) | 30 (88.2%) | 146 (93.0%) |  |  |  |  |
| Between-arm difference in SUV max |  |  |  | <0.001 | 0.047 | 0.091 | < 0.001 |
| - N | 33 | 38 | 160 |  |  |  |  |
| - Median (Range) | 2.60 (-0.30, 17.80) | 0.85 (-0.50, 8.30) | 0.30 (-2.00, 19.30) |  |  |  |  |
| Dose |  |  |  | <0.001 | 0.845 | < 0.001 | < 0.001 |
| - 1 | 14 (42.4%) | 17 (44.7%) | 26 (16.2%) |  |  |  |  |
| - 2 | 19 (57.6%) | 21 (55.3%) | 134 (83.8%) |  |  |  |  |
| Maker |  |  |  | 0.023 | 0.033 | 0.007 | 0.906 |
| - MODERNA | 20 (60.6%) | 13 (35.1%) | 94 (59.5%) |  |  |  |  |
| - PFIZER | 13 (39.4%) | 24 (64.9%) | 64 (40.5%) |  |  |  |  |
| Symptoms2 |  |  |  | 0.068 | 0.335 | 0.373 | 0.020 |
| - No | 28 (84.8%) | 35 (92.1%) | 153 (95.6%) |  |  |  |  |
| - Yes | 5 (15.2%) | 3 (7.9%) | 7 (4.4%) |  |  |  |  |
| Blood Sugar |  |  |  | 0.081 | 0.024 | 0.108 | 0.224 |
| - N | 31 | 36 | 158 |  |  |  |  |
| - Median (Range) | 106.00 (76.00, 202.00) | 101.50 (80.00, 150.00) | 103.00 (68.00, 213.00) |  |  |  |  |
| SUV/blood sugar |  |  |  | 0.003 | 0.099 | 0.104 | 0.001 |
| - N | 31 | 36 | 158 |  |  |  |  |
| - Median (Range) | 0.03 (0.00, 0.20) | 0.02 (0.00, 0.11) | 0.01 (0.00, 0.29) |  |  |  |  |
| Pairwise comparison p value was considered statistically significant when <0.017 based on Bonferroni correction for multiple comparison | | | | | | | |

# Supplemental Table 1B. Vaccine data and between-arm difference in SUV max by days from vaccine

| Var.1 | 0-7 (N=33) | 8-28 (N=120) | >28 (N=78) | p value | p value 0-7 vs 8-28 days | p value 8-28 vs >28 days | p value 0-7 vs >28 days |
| --- | --- | --- | --- | --- | --- | --- | --- |
| Age |  |  |  | 0.370 | 0.947 | 0.180 | 0.336 |
| - N | 33 | 120 | 78 |  |  |  |  |
| - Median (Range) | 69.00 (27.00, 90.00) | 70.00 (20.00, 94.00) | 72.00 (38.00, 91.00) |  |  |  |  |
| Gender |  |  |  | 0.355 | 0.920 | 0.161 | 0.373 |
| - F | 17 (51.5%) | 63 (52.5%) | 33 (42.3%) |  |  |  |  |
| - M | 16 (48.5%) | 57 (47.5%) | 45 (57.7%) |  |  |  |  |
| Race |  |  |  | 0.345 | 0.143 | 0.571 | 0.363 |
| - Non-white | 5 (15.2%) | 8 (7.0%) | 7 (9.2%) |  |  |  |  |
| - White | 28 (84.8%) | 107 (93.0%) | 69 (90.8%) |  |  |  |  |
| Between-arm difference in SUV max |  |  |  | < 0.001 | 0.002 | 0.141 | < 0.001 |
| - N | 33 | 120 | 78 |  |  |  |  |
| - Median (Range) | 2.60 (-0.30, 17.80) | 0.40 (-2.00, 14.90) | 0.20 (-0.60, 19.30) |  |  |  |  |
| Dose |  |  |  | < 0.001 | 0.289 | < 0.001 | < 0.001 |
| - 1 | 14 (42.4%) | 39 (32.5%) | 4 (5.1%) |  |  |  |  |
| - 2 | 19 (57.6%) | 81 (67.5%) | 74 (94.9%) |  |  |  |  |
| Maker |  |  |  | 0.009 | 0.142 | 0.003 | 0.456 |
| - MODERNA | 20 (60.6%) | 54 (46.2%) | 53 (67.9%) |  |  |  |  |
| - PFIZER | 13 (39.4%) | 63 (53.8%) | 25 (32.1%) |  |  |  |  |
| Symptoms2 |  |  |  | 0.048 | 0.122 | 0.198 | 0.013 |
| - No | 28 (84.8%) | 112 (93.3%) | 76 (97.4%) |  |  |  |  |
| - Yes | 5 (15.2%) | 8 (6.7%) | 2 (2.6%) |  |  |  |  |
| Blood Sugar |  |  |  | 0.061 | 0.044 | 0.074 | 0.532 |
| - N | 31 | 117 | 77 |  |  |  |  |
| - Median (Range) | 106.00 (76.00, 202.00) | 102.00 (73.00, 182.00) | 104.00 (68.00, 213.00) |  |  |  |  |
| SUV/blood sugar |  |  |  | 0.004 | 0.013 | 0.162 | < 0.001 |
| - N | 31 | 117 | 77 |  |  |  |  |
| - Median (Range) | 0.03 (0.00, 0.20) | 0.01 (0.00, 0.20) | 0.01 (0.00, 0.29) |  |  |  |  |
| Pairwise comparison p value was considered statistically significant when <0.017 based on Bonferroni correction for multiple comparison | | | | | | | |

# Supplemental Table 2. Univariable linear model predicting between-arm difference in SUV max

| label | estimate95CI | pvalue |
| --- | --- | --- |
| Age | -0.03 ( -0.05 , 0 ) | 0.0676 |
| Gender F | 1.01 ( 0.29 , 1.73 ) | 0.0063 |
| Race Non-white | 2.17 ( 1 , 3.34 ) | <0.001 |
| Daycat 0-7 vs no vaccine | 3.91 ( 2.52 , 5.3 ) | <0.001 |
| Daycat 8-14 vs no vaccine | 1.68 ( 0.33 , 3.02 ) | 0.0150 |
| Daycat >14 vs no vaccine | 1.12 ( 0.03 , 2.21 ) | 0.0442 |
| Daycat2 0-7 vs no vaccine | 3.91 ( 2.52 , 5.3 ) | <0.001 |
| Daycat2 8-28 vs no vaccine | 1.37 ( 0.25 , 2.49 ) | 0.0167 |
| Daycat2 >28 vs no vaccine | 1 ( -0.18 , 2.19 ) | 0.0959 |
| Symptoms2 Yes | -0.04 ( -1.62 , 1.53 ) | 0.9570 |
| Blood Sugar | -0.01 ( -0.03 , 0 ) | 0.0843 |

# Supplemental Table 3A. Multivariable linear model predicting between-arm difference in SUV max

| Term | Beta95CI | pvalue |
| --- | --- | --- |
| Age | -0.02 ( -0.05 , 0.01 ) | 0.1107 |
| GenderF | 0.85 ( 0.18 , 1.51 ) | 0.0131 |
| RaceNon-white | 2.05 ( 0.95 , 3.15 ) | <0.001 |
| Daycat0-7 vs no vaccine | 4.72 ( 3.31 , 6.12 ) | <0.001 |
| Daycat8-14 vs no vaccine | 1.9 ( 0.52 , 3.28 ) | 0.0074 |
| Daycat>14 vs no vaccine | 1.77 ( 0.65 , 2.88 ) | 0.0021 |
| Symptoms2Yes | -0.76 ( -2.17 , 0.65 ) | 0.2921 |
| Blood Sugar | -0.02 ( -0.03 , 0 ) | 0.0466 |

# Supplemental Table 3B. Multivariable linear model predicting between-arm difference in SUV max

| Term | Beta95CI | pvalue |
| --- | --- | --- |
| Age | -0.02 ( -0.05 , 0.01 ) | 0.1122 |
| GenderF | 0.84 ( 0.18 , 1.51 ) | 0.0140 |
| RaceNon-white | 2.06 ( 0.96 , 3.16 ) | <0.001 |
| Daycat20-7 vs no vaccine | 4.72 ( 3.31 , 6.12 ) | <0.001 |
| Daycat28-28 vs no vaccine | 1.82 ( 0.67 , 2.96 ) | 0.0021 |
| Daycat2>28 vs no vaccine | 1.76 ( 0.56 , 2.95 ) | 0.0045 |
| Symptoms2Yes | -0.76 ( -2.17 , 0.66 ) | 0.2950 |
| Blood Sugar | -0.02 ( -0.03 , 0 ) | 0.0450 |

# Supplemental Figure 1. Betweem-arm difference in SUV max stratified by gender


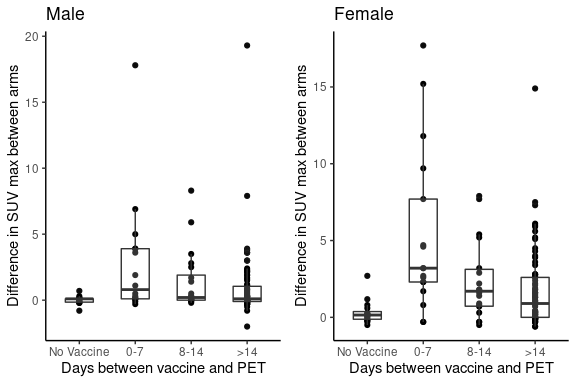


# Supplemental Figure 2. Between-arm difference in SUV max stratified by race


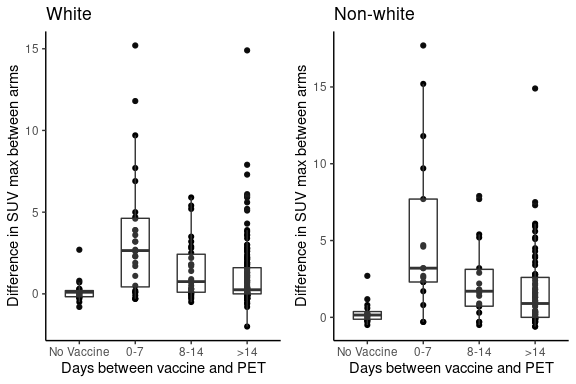

Supplement: Supplementary file 1 — Supplementary file1 (DOCX 59 KB) [file 12149_2021_1675_MOESM1_ESM.docx]
